# Supplementary material for: Multi-scale mapping along the auditory hierarchy using high-resolution functional UltraSound in the awake ferret
Source: eLife. 2018 Jun 28;7:e35028. doi: 10.7554/eLife.35028 (PMC6039176; doi:10.7554/eLife.35028)
Supplement: Transparent reporting form [file elife-35028-transrepform.pdf]

eLIFE
1st Floor
24 Hills Road
Cambridge CB2 1JP, UK
P 01223 855340
W elifesciences.org
T @elife

eLife's transparent reporting form

We encourage authors to provide detailed information *within their submission* to facilitate the interpretation and replication of experiments.Authors can upload supporting documentation to indicate the use of appropriate reporting guidelines for health-related research (see EQUATOR Network), life science research (see the BioSharing Information Resource), or the ARRIVE guidelines for reporting work involving animal research.Where applicable, authors should refer to any relevant reporting standards documents in this form.

If you have any questions, please consult our Journal Policies and/or contact us:editorial@elifesciences.org.

Sample-size estimation

- You should state whether an appropriate sample size was computed when the study was being designed
- You should state the statistical method of sample size computation and any required assumptions
- If no explicit power analysis was used, you should describe how you decided what sample (replicate) size (number) to use

Please outline where this information can be found within the submission (e.g., sections or figure legends), or explain why this information doesn't apply to your submission:

eLife Sciences Publications, Ltd is a limited liability non-profit non-stock corporation incorporated in the State of Delaware, USA, with company number 5030732, and is registered in the UK with company number FC030576 and branch number BR015634 at the address 1st Floor, 24 Hills Road, Cambridge CB2 1JP | August 2014

Tonotopy experiments

The minimum number of trials was computed beforehand with a standardized difference *d* of 1.5 (difference of auditory pixel responses across frequencies in %CBV: 30%; population standard deviation: 20%).

This brings a required number of trials of 7 for reaching an 80% power using a cutoff for statistical significance of 0.05:

$N = 2/ d^2 \times c_{p,power} = 2/d^2 \times c_{0.05,0.80}$

$N = 2/1.5^2 \times 7.9$

N ~ 7 trials

We thus used 10 trials, as a tradeoff between statistical power and recording time (Methods, Protocol for sensory response acquisition). To increase the functional resolution of the technique, we used 20 trials for fig. 2b (Methods, Resolution Quantification).

For the tonotopy experiments, we used at least two ferrets for each region: 3 ferrets (4 craniotomies) for the auditory cortex, 2 ferrets for the MGB, IC and LL (Methods, Animal preparation).

Stimulation experiments

Here, the expected effect was lower: standardized difference *d* of 1.0 (expected evoked activity by stimulation: 20%; population standard deviation: 20%).

$N = 2/ d^2 \times c_{p,power} = 2/d^2 \times c_{0.05,0.80}$

$N = 2/1.0^2 \times 7.9$

N ~ 16 trials

Thus, we used 30 trials (Methods, FC stimulation).

In that case, we used one animal for the fUS connectivity experiment and one animal for the tracer injection, thus raising to two the number of animals used (Methods, FC stimulation/Anatomical tracers). As it was mainly a proof of concept, we believe that two animals (stimulation experiments being consistently performed several times, across days, in the same animal) with converging results, by two different methods, is a sufficient number.

Replicates

- You should report how often each experiment was performed
- You should include a definition of biological versus technical replication
- The data obtained should be provided and sufficient information should be provided to indicate the number of independent biological and/or technical replicates
- If you encountered any outliers, you should describe how these were handled
- Criteria for exclusion/inclusion of data should be clearly stated
- High-throughput sequence data should be uploaded before submission, with a private link for reviewers provided (these are available from both GEO and ArrayExpress)

Please outline where this information can be found within the submission (e.g., sections or figure legends), or explain why this information doesn't apply to your submission:

Tonotopy experiments were replicated in at least two ferrets (biological replicates) (Methods, Animal preparation). Intriguingly, two additional ferrets did not show any reliable response to sound (responses below 10%CBV), for unknown reasons. They were not used in the experiments (Methods, Signal processing, analysis & statistics).
Stimulation experiments were performed in one ferret, and tracer injections in one ferret (biological replicates) (Methods, FC stimulation/Anatomical tracers).

Statistical reporting

- Statistical analysis methods should be described and justified
- Raw data should be presented in figures whenever informative to do so (typically when N per group is less than 10)
- For each experiment, you should identify the statistical tests used, exact values of N, definitions of center, methods of multiple test correction, and dispersion and precision measures (e.g., mean, median, SD, SEM, confidence intervals; and, for the major substantive results, a measure of effect size (e.g., Pearson's r, Cohen's d)
- Report exact p-values wherever possible alongside the summary statistics and 95% confidence intervals. These should be reported for all key questions and not only when the p-value is less than 0.05.

Please outline where this information can be found within the submission (e.g., sections or figure legends), or explain why this information doesn't apply to your submission:

<u>Tuning curve</u>
One-way ANOVA with post-hoc Wilcoxon rank sum test for each frequency pair was used to test the significance of the frequency-dependent voxel responses (Methods, Methods, Signal processing, analysis & statistics).

<u>Decoding analysis</u>
We used a permutation procedure, with 100 permutations, thus allowing us to quantify a p-value with resolution 0.01. All actual decoding accuracies were out of the chance distribution (Methods, Signal processing, analysis & statistics).

<u>Depth decoding analysis</u>
One-way repeated-measure ANOVA across craniotomies was used to assess the effect of depth (Methods, Signal processing, analysis & statistics).

<u>Functional resolution</u>
Two-way ANOVA. P-values were not used here (only the voxel and interaction – voxel x frequency – factors). Instead randomization was used to estimate significance ($p<5e-2$, 5% percentile over 50 randomizations) (Methods, Signal processing, analysis & statistics).

All errorbars shown are mean±2sem (fig 3b, and fig 3 supplements 1 and 3), except for fig. 1c (mean±sem for clarity).
All statistical analysis was performed using Matlab (The Mathworks, Natick).

(For large datasets, or papers with a very large number of statistical tests, you may upload a single table file with tests, Ns, etc., with reference to sections in the manuscript.)

Group allocation

- Indicate how samples were allocated into experimental groups (in the case of clinical studies, please specify allocation to treatment method);if randomization was used, please also state if restricted randomization was applied
- Indicate if masking was used during group allocation, data collection and/or data analysis

Please outline where this information can be found within the submission (e.g., sections or figure legends), or explain why this information doesn't apply to your submission:

NA

Additional data files ("source data")

- We encourage you to upload relevant additional data files, such as numerical data that are represented as a graph in a figure, or as a summary table
- Where provided, these should be in the most useful format, and they can be uploaded as "Source data" files linked to a main figure or table
- Include model definition files including the full list of parameters used
- Include code used for data analysis (e.g., R, MatLab)
- Avoid stating that data files are "available upon request"

Please indicate the figures or tables for which source data files have been provided:

NA
